# Stereotactic Radiosurgery With Concurrent Immunotherapy in Melanoma Brain Metastases Is Feasible and Effective

**DOI:** 10.3389/fonc.2020.592796

**Published:** 2020-10-15

**Authors:** Jakob Liermann, Julia K. Winkler, Mustafa Syed, Ulf Neuberger, David Reuss, Semi Harrabi, Patrick Naumann, Jonas Ristau, Fabian Weykamp, Rami A. El Shafie, Laila König, Jürgen Debus, Jessica Hassel, Stefan Rieken

**Affiliations:** ^1^Department of Radiation Oncology, Heidelberg University Hospital, Heidelberg, Germany; ^2^Heidelberg Institute of Radiation Oncology (HIRO), Heidelberg, Germany; ^3^Heidelberg Ion-Beam Therapy Center (HIT), Heidelberg, Germany; ^4^National Center for Tumor Diseases (NCT), Heidelberg, Germany; ^5^Department of Dermatology, Heidelberg University Hospital, Heidelberg, Germany; ^6^Department of Neuroradiology, Heidelberg University Hospital, Heidelberg, Germany; ^7^Heidelberg University Hospital, Institute of Pathology, Heidelberg, Germany; ^8^Clinical Cooperation Unit Radiation Oncology, German Cancer Research Center (DKFZ), Heidelberg, Germany; ^9^German Cancer Consortium (DKTK), Partner Site Heidelberg, German Cancer Research Center (DKFZ), Heidelberg, Germany

**Keywords:** stereotactic radiotherapy (SRT), radiosurgery (SRS), melanoma, brain metastases (BM), radiation necrosis (RN), immunotherapy

## Abstract

**Objective:** Stereotactic radiosurgery (SRS) is an established treatment for brain metastases in the management of metastasized melanoma. The increasing use of checkpoint inhibitors in melanoma therapy leads to combined treatment schemes consisting of immunotherapy and SRS that need to be evaluated regarding safety and feasibility.

**Methods:** We retrospectively analyzed 36 patients suffering from cerebral metastasized melanoma. Between November 2011 and May 2016, altogether 66 brain metastases were treated with single-fraction SRS (18–20 Gy prescribed to the 80% isodose) in combination with a checkpoint inhibitor (ipilimumab: 82%, pembrolizumab: 14% or nivolumab: 4%), administered within 3 months before or after SRS. Toxicity was evaluated with focus on the incidence of central nervous system (CNS) radiation necrosis (CRN). Overall survival (OS), freedom from local progression (FFLP), freedom from central nervous system radiation necrosis (FFCRN), and freedom from distant intracranial progression (FFDIP) were analyzed using the Kaplan-Meier method.

**Results:** The median follow-up was 25 months (range: 2–115 months). Two patients (6%) presented with cerebral edema CTCAE °III and another two patients (6%) presented with one-sided muscle weakness CTCAE °III after SRS. One of these four symptomatic cases correlated with an observed CRN, the other three symptomatic cases were related to local tumor progression (*n* = 2) or related to the performance of additional whole brain radiotherapy (WBRT). No further CTCAE °III or °IV toxicity was seen. During follow-up, seven of the growing contrast-enhanced lesions were resected, revealing two cases of CRN and five cases of local tumor progression. Altogether, the observed CRN rate of the irradiated metastases was 6–17% at the time of analysis, ranging due to the radiologically challenging differentiation between CRN and local tumor progression. The observed ranges of the 1- and 2-years FFLP rates were 82–85% and 73–80%, respectively. The median FFDIP was 6.1 months, the median OS was 22.2 months.

**Conclusion:** In the presented cohort, the combination of SRS and checkpoint inhibitors in the management of cerebral metastasized melanoma was safe and effective. Compared to historic data on SRS only, the observed CRN rate was acceptable. To gain resilient data on the incidence of CRN after combined treatment schemes, prospective trials are needed.

## Introduction

Prior to the era of immunotherapy and targeted therapy in metastasized melanoma, newly diagnosed brain metastases (BM) came along with a drastic decline of survival probability with an observed median overall survival (OS) of 4–5 months (1, 2). The emergence of immunotherapy and targeted therapy such as PD-1 antibodies or BRAF inhibitors resulted in fundamentally improved oncological outcomes (3–9). Furthermore, a potential synergistic effect of combined therapy regimens of stereotactic radiosurgery (SRS) and checkpoint inhibitors was observed by Lehrer et al. in a meta-analysis of 17 studies (10). The recent median OS of melanoma patients with BM is 14–23 months (11, 12).

Due to the prolonged survival times, there is an increasing number of patients suffering from BM that are treated by SRS and concurrent immunotherapy. Hence, long-term toxicity of RT is gaining more interest. There is an ongoing discussion on safety of combined treatment schemes of immunotherapy or targeted therapy with SRS since the central nervous system (CNS) radiation necrosis (CRN) risk could potentially be increased, as described in several analyses (13, 14).

There is no clear radiological definition of CRN and incidence rates differ drastically in different studies based on the underlying diagnostic criteria (15). Several studies aimed to implement effective diagnostic procedures (16–19), but in clinical routine, we are regularly confronted with missing perfusion sequences in the presented MRI images, absence of PET-imaging or other causes of uncertainty (e.g., inconsistent results or atypical courses of the disease). However, even in interdisciplinary decision-making, the differential diagnosis of tumor progression and CRN remains challenging and the accuracy of diagnosis remains uncertain.

In this study, we evaluated toxicity and survival outcomes of 36 melanoma patients with BM that received SRS with concurrent immune checkpoint inhibitors at our institution.

## Patients and Methods

### Patients

In the present retrospective analysis, we included melanoma patients with BM, that were treated with SRS and concurrent immunotherapy with checkpoint inhibitors. Previous studies have defined concurrent therapy as application of immunotherapy within 1–4 months of SRS (20, 21). In the present study, concurrent immunotherapy was defined within 3 months before or after SRS. Treatments were performed from November 2011 to May 2016 at our institution. Clinical information was extracted from the charts. As described previously by Sperduto et al., the graded prognostic assessment score for melanoma using molecular markers (Melanoma-molGPA) was evaluated for each patient (22).

### Radiotherapy

RT was performed as linear accelerator-based single-fraction SRS with photons with an energy of 6 MeV. Total doses of 18–20 Gy were prescribed to the 80% isodose line. Patients were positioned in an individually manufactured scotch-cast mask to effectively reduce movements to a maximum of 1–2 mm (23). The mask was attached to an external stereotactic localization system. Contrast-enhanced CT scans and contrast-enhanced MRI imaging with slice thicknesses of no more than 3 mm were performed as planning imaging. The gross tumor volume (GTV) was defined as the contrast-enhanced visible tumor in the axial T1-weighted MRI sequence. For planning uncertainties, a margin of 2–3 mm was added to define the planning target volume (PTV). Irradiation was done with multileaf collimators with a leaf width of 1.5 mm at the isocenter. Before and after SRS, dexamethasone therapy was administered to avoid acute radiation toxicity in form of increased intracranial pressure.

### Immunotherapy

Immunotherapy was applied intravenously. Ipilimumab was administered in doses of 3 mg/kg body weight every 3 weeks for up to four cycles. The dose of pembrolizumab was set at 2 mg/kg body weight every 3 weeks. Nivolumab was applied in doses of 3 mg/kg body weight in 2-weeks intervals. Immunotherapy was defined as concurrent when administered up to 3 months before or after SRS.

### Follow-Up

Follow-up time was defined from the day of SRS until last clinical evaluation or MRI scan. Follow-up was performed at least every 3 months by contrast-enhanced MRI scans and clinical evaluation. For response evaluation, we decided to define local tumor progression through interdisciplinary decision of dermatologists, neuroradiologists and radiation oncologists, whenever the clinical diagnoses seemed reliable. Nevertheless, in some cases there were still doubts about the differentiation between CRN or local tumor progression. In the present study, these uncertain cases were evaluated separately as described in the following paragraph.

### Local Tumor Progression and Incidence of CRN

One of the main challenges is to differentiate CRN and local tumor progression. In some cases, resection was performed. Of all resected lesions, we double-checked the original histological findings of these lesions by an uninvolved neuropathologist who was instructed to differentiate between CRN and vital tumor. Additionally, there were some cases without histological results that were diagnosed by interdisciplinary decision and in which the diagnoses seemed reliable (e.g., when distant intracranial metastases were rapidly growing in the same manner and at the same time as the irradiated lesion). Combining these cases of resected and unresected lesions with reliable clinical diagnosis, we could define the minimal rate of the observed local tumor progression. As definition of the maximum rate, all interdisciplinary diagnosed but uncertain cases of CRN vs. local tumor progression were counted as local tumor progression. Using these minimum and maximum rates, we were able to define a range of the actual local tumor progression rate. In a second step, we assumed the interdisciplinary diagnosis of the uncertain cases to be the most probable diagnosis and calculated freedom from local progression (FFLP) and freedom from CNS radiation necrosis (FFCRN), accordingly (“estimated” results). FFLP and FFCRN were defined from the day of SRS until last clinical evaluation/last MRI scan or date of local tumor progression or CRN. Each lesion was evaluated independently (*n* = 66).

Additionally, we double-checked pre-resection MRI-based interdisciplinary diagnosis of the histologically confirmed cases blinded and separately by a neuroradiologist and a radiation oncologist to finally get an idea about the accuracy of the interdisciplinary diagnosis made in clinical routine.

### Lesion Size Evaluation in the Course of the Disease

Another aim of the present study was to analyze size development of the irradiated BM not only for tumor response evaluation, but also for analyzing potential differences in the character of CRN and local tumor progression. For this purpose, the largest diameter of the irradiated lesions (contrast-enhanced T1-weighted sequence) and the largest diameter of the surrounding edema (T2-weighted sequence, mostly fluid attenuated inversion recovery (FLAIR) sequence) were measured with two perpendicular diameters in the planning MRI scan as well as in each follow-up MRI scan, respectively. Relative and absolute size development was compared.

### Distant Intracranial Tumor Progression

Distant intracranial tumor progression was defined as occurrence of new BM in the follow-up MRI scans. It was defined from the day of SRS until last clinical evaluation/last MRI scan or date of first description of distant intracranial tumor progression in MRI imaging. Freedom from distant intracranial progression (FFDIP) was calculated based on each irradiated lesion (*n* = 66).

### Overall Survival

OS after SRS was defined from the day of the first SRS until last clinical evaluation/last MRI scan or date of death. OS after initial diagnosis of BM was defined from the date of initial diagnosis of BM until last clinical evaluation or date of death. Each patient was evaluated (*n* = 36).

### Toxicity

Disruptions of the blood-brain barrier were defined as new contrast-enhancement (24) within the radiation field without typical signs of CRN or local tumor progression. Often, perifocal edema could also be observed (25). CNS toxicity rates after SRS were extracted from the charts and graded according to the Common Terminology Criteria for Adverse Events (CTCAE) in the version 4.03.

### Statistics

OS, FFLP, FFCRN, and FFDIP were analyzed using the Kaplan-Meier method. Statistics and figures were performed with GraphPad Prism 8.2.1 (GraphPad Software, La Jolla, CA, USA).

### Ethics

The study was approved by the ethics committee of the University of Heidelberg, Germany (S-172/2018).

## Results

### Baseline Characteristics

After a median follow-up of 25 months (range 2–115 months), 66 brain metastases of 36 patients were analyzed. The patient cohort included 28 men and eight women. The median age at time of initial diagnosis of BM was 63 years (range 36–81 years). The median time from initial diagnosis of the melanoma until development of BM was 2.6 years (range 0–41 years). Karnofsky performance status scale (KPS) and Melanoma-molGPA score are shown in Table 1. The median L-lactate dehydrogenase (LDH) level in the most recent blood sample before SRS was 203 U/L (range 115–815 U/L, reference value up to 215 U/l). The main locations of the supposed underlying primary melanoma were the skin of the back, followed by the head and unknown primary locations. Thirty-nine percent of the patients presented with BRAF-mutations (mostly V600 E), in 58% of the patients there was no BRAF-mutation and in one patient BRAF-mutations were not tested.

**Table 1:** Baseline characteristics.

	***n***	**(%)**
**Number of patients**	**36**	**(100)**
Sex		
Male	28	(78)
Female	8	(22)
Age at initial diagnosis of BM (median, range)	63 y (36–81)	
Karnofsky Performance Status Scale		
90–100%	18	(50)
80%	10	(28)
70%	2	(5.5)
60%	2	(5.5)
Unknown	4	(11)
Melanoma-molGPA Score		
0.0–1.0	3	(8.5)
1.5–2.0	16	(44.5)
2.5–3.0	12	(33)
Unknown	5	(14)
LDH level before SRS (median, range)	203 U/L (115–815)	
Site of Primary Tumor		
Back	9	(25)
Breast	3	(8)
Abdomen	1	(3)
Foot	4	(11)
Hand	1	(3)
Head and Neck	6	(16.5)
Arm	4	(11)
Leg	2	(6)
Unknown	6	(16.5)
WBRT		
Before SRS	3	(8)
After SRS	9	(25)
**Number of irradiated BM**	**66**	**(100)**
BRAF		
Mutation	14	(39)
No mutation	21	(58)
Unknown	1	(3)
Time from initial diagnosis of melanoma to BM (median, range)	2.6 y (0–41)	
Immunotherapy		
Ipilimumab 3 mg/kg body weight	54	(81)
Pembrolizumab 2 mg/kg body weight	9	(14)
Nivolumab 3 mg/kg body weight	3	(5)
Single-Fraction SRS		
Total dose: 20 Gy to 80% isodose line	53	(80)
Total dose: 18 Gy to 80% isodose line	13	(20)
PTV volume (median, range)	1.3 cm^3^ (0.4–10.7)	
Longest diameter of BM in contrast-enhanced MRI (median, range)	7.5 mm (4–23)	
BM Location		
Frontal	21	(32)
Temporal	19	(29)
Occipital	5	(7.5)
Parietal	9	(13.5)
Cerebellar	12	(18)

Single-fraction SRS was applied with a total radiation dose of 20 Gy to the 80% isodose line in 53 cases. In 13 cases, a total dose of 18 Gy to the 80% isodose line was prescribed. In 86% of all cases, radiation plans with eight radiation fields were delivered. In 11 and 3% of the cases, seven fields and nine fields were used, respectively. The median PTV volume was 1.3 cm^3^ (range 0.4–10.7 cm^3^). All patients received dexamethasone before and after SRS, in 68% of the cases delivered orally as 8 mg tablets 1 h before and 6 h after radiosurgery. Alternatively, patients were treated with 20 mg dexamethasone intravenously 1 h before and 6 h after radiosurgery (8%) or with 8 mg dexamethasone tablets 1 h before, 6 h after, and ~12 h after SRS (24%). Three patients were treated with whole brain radiotherapy (WBRT) before SRS and nine patients received WBRT in the course of their disease, after SRS.

The most commonly used concurrent immunotherapy was ipilimumab in 81% of all cases, followed by pembrolizumab (14%) and nivolumab (5%).

At the time of SRS, the largest diameter of the BM was at a median of 7.5 mm (range 4–23 mm), measured on axial T1-weighed contrast-enhanced MRI sequences. BM were mainly located in one of the frontal lobes (32%) or in one of the temporal lobes (29%) and rarely in the cerebellum (8%). In the course of the disease, 11% of the irradiated lesions were resected after SRS.

Baseline patient and BM characteristics are summarized in Table 1.

### Local Tumor Progression

**Figure 1:**
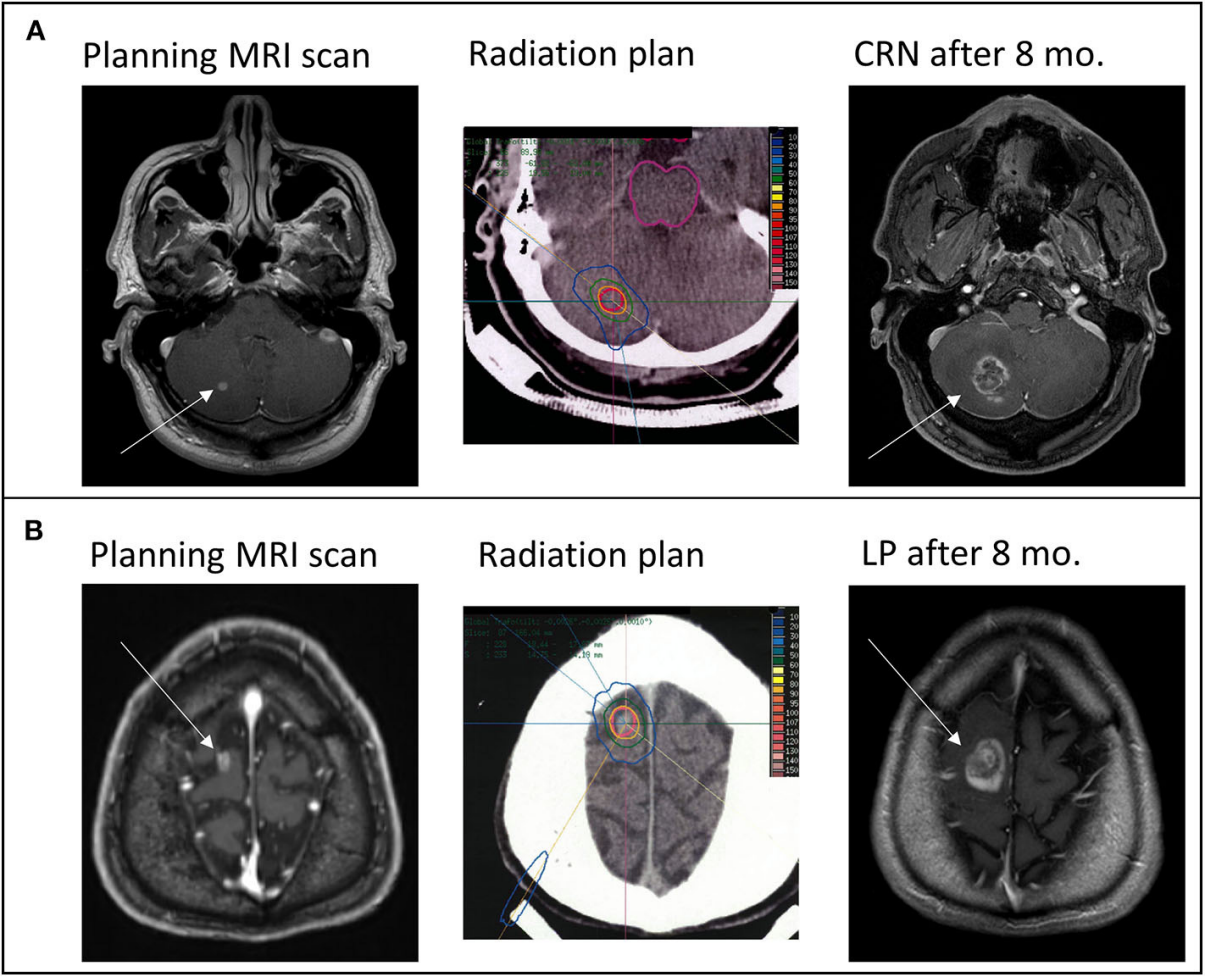
From left to right: Pre-treatment T1-weighted axial contrast-enhanced Magnetic resonance imaging (MRI) sequence showing new diagnosed brain metastasis (white arrow), representative slice of the irradiated radiation plan showing isodose lines (red = 100% isodose line) in the planning Computed tomography (CT) and follow up T1-weighted axial contrast-enhanced MRI sequence of **(A)** an histological confirmed CNS radiation necrosis (CRN) and **(B)** an histological confirmed local progression (LP) ~8 months (mo.) after combined treatment of checkpoint-inhibition and stereotactic radiotherapy.

Seven BM were resected in the course of the disease, showing two cases of CRN and five cases of local tumor progression (Figure 1). Additionally, there were six cases of clinically diagnosed reliable local tumor progression. Consequently, the minimum local tumor progression rate at the time of analysis was 17% (*n* = 11). Seven clinical diagnoses were deemed uncertain. The maximum local tumor progression rate at the time of analysis consequently was 27% (*n* = 18). The ranges of the 1-, 2-, and 5-years FFLP were 82–85%, 73–80%, and 62–80%, respectively.

**Figure 2:**
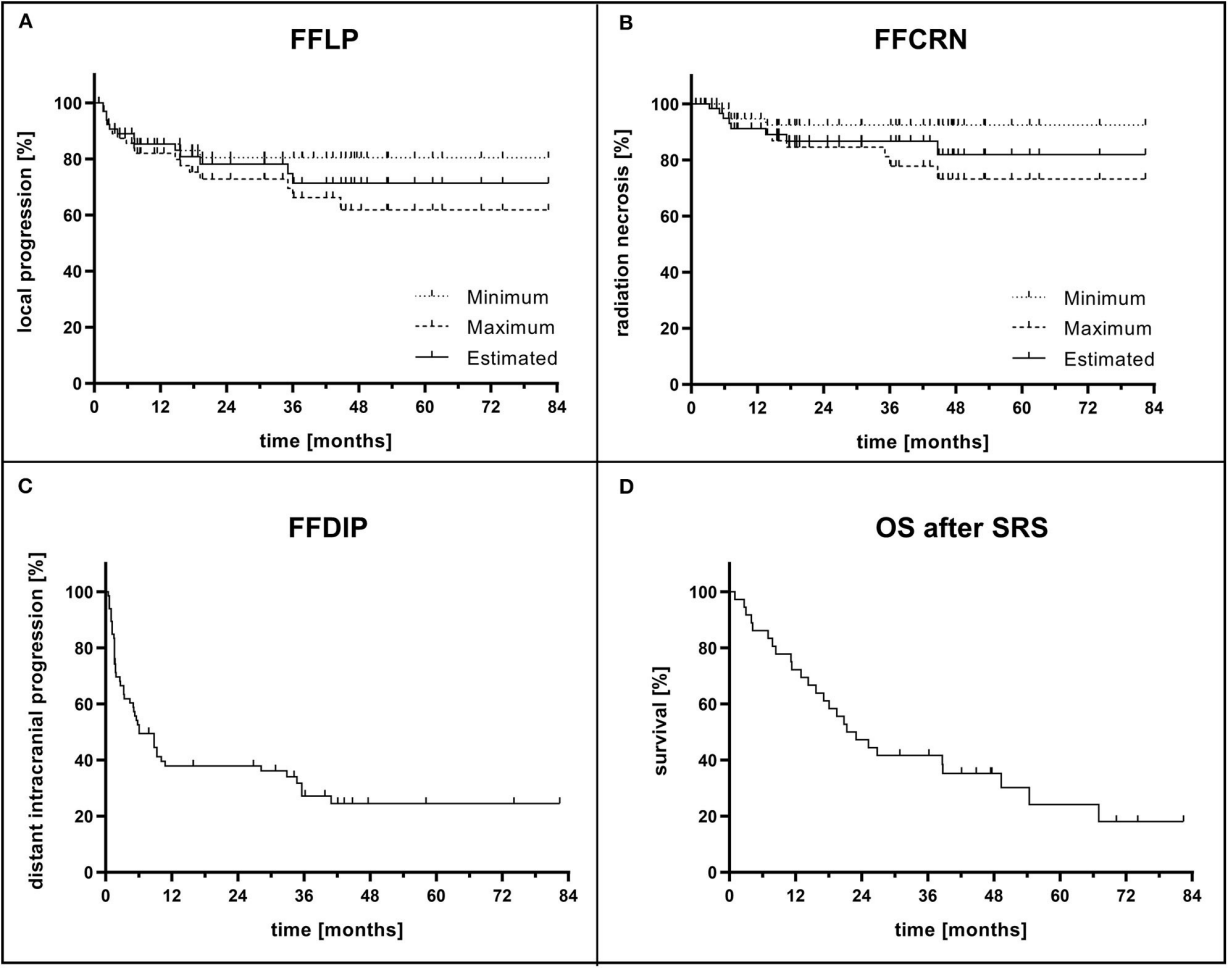
**(A)** Freedom from local progression (FFLP) and **(B)** freedom from CNS radiation necrosis (FFCRN) after single-fraction stereotactic radiosurgery (SRS) and concurrent immunotherapy calculated for each brain metastasis (BM, *n* = 66). The actual rates are expected to be in the range between minimum and maximum rate. Based on interdisciplinary decision, the estimated result is shown. **(C)** Freedom from distant intracranial progression (FFDIP) after SRS and concurrent immunotherapy calculated for each BM. **(D)** Overall survival (OS) after SRS and concurrent immunotherapy calculated for each patient (*n* = 36).

Out of the seven uncertain cases, three were clinically diagnosed as rather being local tumor progression and four as rather being CRN by interdisciplinary consent. Considering these assumptions, the estimated FFLP rates at 1-, 2-, and 5 years were 85, 78, and 71% (Figure 2A).

Re-evaluation of the histologically proven lesions showed that clinical diagnosis accuracy of local tumor progression was 80% (retrospectively, in four of the five lesions the clinical diagnosis was correct). Clinical diagnosis accuracy of CRN was 50% (retrospectively, in one of the two lesions the clinical diagnosis was correct). Altogether, in five of the seven cases, the interdisciplinary diagnosis was correct (71%).

### Incidence of CNS Radiation Necrosis

In addition to the two histologically proven cases of CRN, two clinically diagnosed cases of CRN could be observed, that were reliable due to their course of disease. As described above, the minimum CRN rate at the time of analysis was consequently 6% (*n* = 4). Counting the seven uncertain clinical diagnoses as CRN, the maximum CRN rate at the time of analysis was 17% (*n* = 11). The ranges of the 1-, 2-, and 5-years FFCRN were 91–95%, 85–92%, and 73–92%, respectively. Considering the clinical diagnosis of these uncertain cases, the estimated FFCRN rates at 1-, 2-, and 5 years were 91, 87, and 82% (Figure 2B). The observed CRN occurred mainly in the 1st year after SRS (median: 7.1 months, range: 3.3–44.7 months).

### Distant Intracranial Tumor Progression

Distant intracranial tumor progression occurred in 70% of the cases (*n* = 46 lesions). At the time of analysis, 28% of the patients had not developed distant intracranial tumor progression at all (*n* = 10). In limited tumor progression, SRS was performed as salvage treatment. In more severe cases, WBRT or best supportive care were initiated. The median FFDIP was 6.1 months (Figure 2C). One- and 2-years FFDIP were 38%, each. Five-years FFDIP was 25%.

### Overall Survival

Ten patients (28%) were alive at the time of analysis. The observed median OS after SRS was 22.2 months (Figure 2D). One- and 2-years OS rates were 69 and 44%, respectively. The 5-years OS rate was 18%. The median OS since initial diagnosis of BM was 24.9 months. 1-, 2-, and 5-years OS rates were 78, 50, and 20%, respectively.

### Toxicity

Among the observed CRN, there were two cases that were resected in the course of the disease. One of the two patients presented with headache CTCAE °II. The other patient did not present any symptoms and resection was performed to exclude local tumor progression. Another patient with clinically diagnosed CRN suffered from decreased vision CTCAE °II and presented with a seizure CTCAE °II. Furthermore, the patient described fatigue CTCAE °II and a left-sided muscle weakness CTCAE °III. This patient was successfully treated with Bevacizumab 7.5 mg/kg body weight in 4 cycles, each cycle administered intravenously every 2 weeks, after being previously treated with dexamethasone without benefit. All other cases of CRN were self-limited. Three of these cases were temporarily treated with dexamethasone. In addition to the occurrence of CRN, 10 asymptomatic cases (15%) of disruptions of the blood-brain barrier of the irradiated lesions could be observed. All of them were self-limited without any need of intervention.

A total of 10 cases of hemorrhage of the irradiated BM could be observed (15%). Nine of these were limited with no further intervention needed, one hemorrhage came along with a local tumor progression that was resected. The observed hemorrhages occurred mainly in the 1st year after SRS (median: 4.9 months, range: 1.6–44.7 months).

Six percent of all patients presented with cerebral edema CTCAE °III after SRS. Furthermore, 6% of the patients developed one-sided muscle weakness CTCAE °III. No other higher graded toxicity could be observed. Fourteen percent of all patients suffered from fatigue CTCAE °II and 6% suffered from fatigue CTCAE °I in the course of the disease. Eleven percent of the patients presented with headache CTCAE °I and 6% with headache CTCAE °II. Seizures CTCAE °II could be observed in 6% of the patients. One patient presented with a seizure CTCAE °I. One patient presented dysarthria CTCAE °II and one patient presented decreased vision CTCAE °II. The described symptoms mostly came along with distant intracranial tumor progression or additional WBRT.

### Lesion Size Evaluation in the Course of the Disease

**Figure 3:**
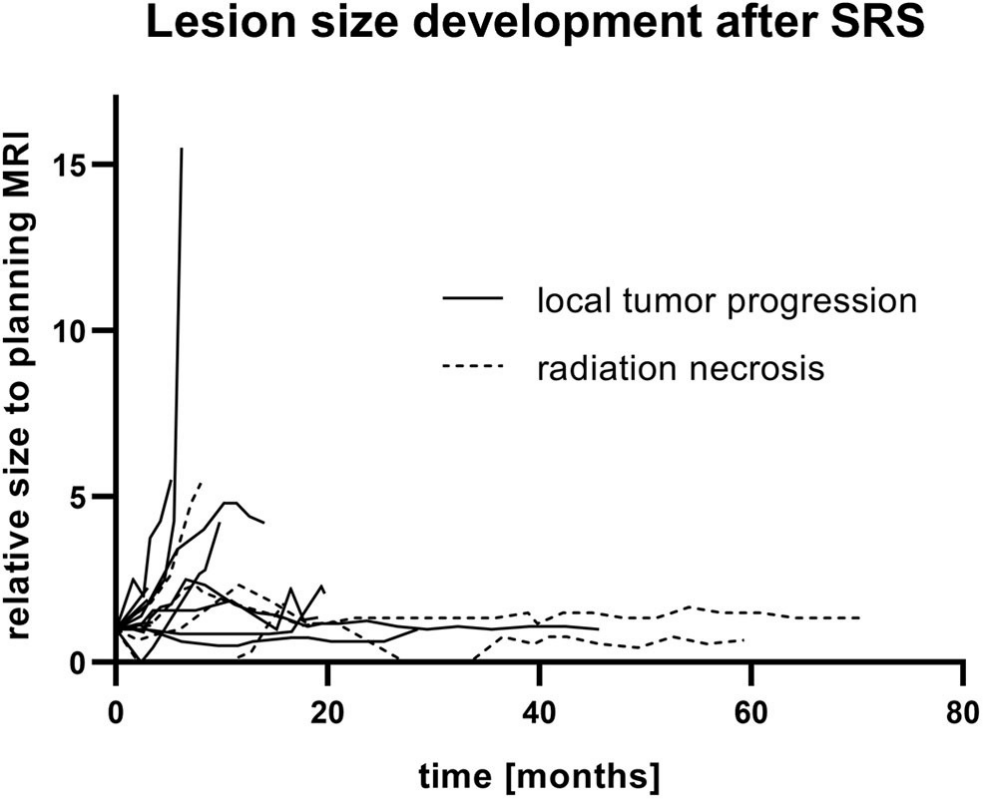
Lesion size development after single-fraction stereotactic radiosurgery (SRS) and concurrent immunotherapy of all reliable diagnoses of local tumor progression and CNS radiation necrosis. Relative change of the biggest diameter in the contrast-enhanced T1-weighted sequence of follow-up MRI to the corresponding planning MRI is shown.

There was no difference between the size development of irradiated lesions in the two groups of local tumor progression and CRN, neither in the measurements of the contrast-enhanced lesions nor in the measurements of the edema. Representative for all analyses, relative changes in the largest diameter of contrast-enhanced lesions of local tumor progression and CRN are shown in Figure 3.

## Discussion

Immunotherapy has drastically improved OS rates in metastasized melanoma patients. Consequently, combined treatment regimens of SRS and checkpoint inhibitors are increasingly performed. Potential risks and benefits of this treatment combination are still being discussed. In the present retrospectively performed analysis of 66 BM, single-fraction SRS and concurrent immunotherapy at our institution seemed safe and effective.

Although this is a retrospective study and the patient cohort is small, the evaluation of Melanoma-molGPA scores revealed a comparable distribution to the patient cohort of Sperduto et al. (22) with slightly worse prognosis of patients in the present study. Thirty-three percent of all patients underwent WBRT either before or after SRS. This high rate of re-irradiation might have influenced outcomes drastically. Still, we decided to include these patients in our analysis because this reflects the genuine patient cohort seen in our institution. Additionally, similar rates of WBRT could be observed in previous studies (26), although the benefit of WBRT is controversial (27, 28). The present analysis represents a homogenous therapy scheme consisting of single-fraction SRS and immunotherapy with checkpoint inhibitors, mainly ipilimumab.

Response evaluation was done through interdisciplinary consent. For response evaluation, there is a recommendation of the Response Assessment in Neuro-Oncology Brain Metastases (RANO-BM) working group (29). According to the RANO-BM recommendation, in the present study, the contrast-enhanced lesions were measured with two perpendicular diameters. Nevertheless, response evaluation could finally not be done as recommended because of two limitations. Firstly, measurable lesions are demanded to have a size of at least 10 mm. In our cohort, just one third of all BM were larger than 10 mm. Twelve percent (*n* = 8) of all metastases were even smaller than 5 mm. Secondly, the main problem in response evaluation is the differentiation between CRN and local tumor progression that is not included in the response evaluation criteria.

The observed FFLP range in the present study of 82–85% after 12 months seems in line with previous data, showing FFLP rates of SRS in patients with melanoma after 12 months at ~60–83% (14, 20, 26, 30–33). In the above-mentioned meta-analysis of Lehrer et al., a local control rate of 89.2% was found (10). Hence, our data demonstrate the effectiveness of stereotactic RT in limited BM in patients suffering from melanoma. The described FFDIP of 38% after 12 and 24 months is worse than the one observed by Hadi et al. who described rates of 54.4 and 36.6% (32) but in line with the meta-analysis of Lehrer et al. reporting 36.8% regional brain control after 12 months (10). The published median overall survival of 15–22 months seems in line with our data (14, 26, 34). Lehrer et al. described an 1-year OS rate of 65% in the combined treatment group (10) which is comparable to the observed 69% in the our patient cohort. The observed median OS of 24.9 months after initial diagnosis of BM in the present study reflects the high OS rates, seen in patients treated by modern therapy schemes (12).

The strengths of this work are the long follow-up and the high rate of resected BM that could help estimate the accuracy of MRI-based differentiation between CRN and local tumor progression which is critical in evaluating RT outcomes of BM. To avoid incorrect pathological reports by unspecified instructions, all resected lesions were re-evaluated by a neuropathologist with the instruction to differentiate between CRN and local tumor progression. In these histologically proven cases, we could demonstrate that the accuracy rate of interdisciplinary decision based on MRI imaging and clinical information is ~70%, highlighting the difficulty of diagnosis in clinical routine.

Despite promising advances in improvement of diagnostic procedures (16–19), it is regularly necessary to decide mainly on the basis of standard MRI imaging and clinical information, as it was the case in the presented cohort. We tried to address this major problem in the judgement of follow-up MRI scans after cerebral RT and intended to evaluate CRN rates including this uncertainty. The reported FFCRN range of 91–95% after 12 months in the present analysis is in line with previously published data, even though the FFCRN rates differ among several studies. After 12 months, Patel et al. and Minniti et al. described CRN rates of 18 and 17%, respectively (20, 31). Hadi et al. reported a FFCRN rate of 82.1% after 12 months (32). Diao et al. and Lehrer et al. reported CRN rates of only 5% (10, 14). Comparing to results of SRS without concurrent immunotherapy with a 1-year CRN rate of 17.2% (35), the CRN rate of the present study does not seem increased.

The improvement of OS rates by immunotherapy has led to the hypothesis that RT of BM could potentially be omitted. Recently, in two studies, combined immunotherapy of nivolumab and ipilimumab was tested as primary therapy of asymptomatic BM (4, 6). Although response rates are promising, toxicity rates were very high with 55% °III or °IV adverse events reported by Tawbi et al. and 54% observed by Long et al. In our analysis, we demonstrated safety and efficacy of combined treatment schemes of SRS and checkpoint inhibitors, supporting the use of this treatment strategy.

In the present study, the combination of SRS and checkpoint inhibitors was safe. Overall survival and CRN rates seem acceptable and are in line with previous data.

## Data Availability Statement

The raw data supporting the conclusions of this article will be made available by the authors, without undue reservation.

## Ethics Statement

The studies involving human participants were reviewed and approved by the ethics committee of the University of Heidelberg, Germany (S-172/2018). Written informed consent for participation was not required for this study in accordance with the national legislation and the institutional requirements.

## Author Contributions

JL, JH, and SR designed and directed the project. JL and JW gathered the data. JL analyzed the data and wrote the manuscript. UN and JL analyzed MR imaging. DR evaluated histological findings. SH, PN, JR, FW, RE, and LK helped finalizing the manuscript. JD, JH, and SR supervised the project. All authors contributed to the article and approved the submitted version.

## Conflict of Interest

JD received grants from Merck Serono GmbH, The Clinical Research Institute GmbH (CRI), View Ray Inc., Accuray Incorporated, RaySearch Laboratories AB, Vision RT limited, Astellas Pharma GmbH, Astra Zeneca GmbH, Solution Akademie GmbH, Ergomed PLC Surrey Research Park, Siemens Healthcare GmbH, Quintiles GmbH, Pharmaceutical Research Associates GmbH, Boehringer Ingelheim Pharma GmbH Co, PTW-Freiburg Dr. Pychlau GmbH, and Nanobiotix A. A. outside the submitted work. The remaining authors declare that the research was conducted in the absence of any commercial or financial relationships that could be construed as a potential conflict of interest.

## References

[1] Davies MA Liu P McIntyre S Kim KB Papadopoulos N Hwu WJ. Prognostic factors for survival in melanoma patients with brain metastases. Cancer. (2011) 117:1687–96. 10.1002/cncr.25634 20960525

[2] Fife KM Colman MH Stevens GN Firth IC Moon D Shannon KF. Determinants of outcome in melanoma patients with cerebral metastases. J Clin Oncol. (2004) 22:1293–300. 10.1200/JCO.2004.08.140 15051777

[3] Davies MA Saiag P Robert C Grob JJ Flaherty KT Arance A. Dabrafenib plus trametinib in patients with BRAF(V600)-mutant melanoma brain metastases (COMBI-MB): a multicentre, multicohort, open-label, phase 2 trial. Lancet Oncol. (2017) 18:863–73. 10.1016/S1470-2045(17)30429-1 28592387 PMC5991615

[4] Long GV Atkinson V Lo S Sandhu S Guminski AD Brown MP Combination nivolumab and ipilimumab or nivolumab alone in melanoma brain metastases: a multicentre randomised phase 2 study. Lancet Oncol. (2018) 19:672–81. 10.1016/S1470-2045(18)30139-6 29602646

[5] Margolin K Ernstoff MS Hamid O Lawrence D McDermott D Puzanov I. Ipilimumab in patients with melanoma and brain metastases: an open-label, phase 2 trial. Lancet Oncol. (2012) 13:459–65. 10.1016/S1470-2045(12)70090-6 22456429

[6] Tawbi HA Forsyth PA Algazi A Hamid O Hodi FS Moschos SJ. Combined nivolumab and ipilimumab in melanoma metastatic to the brain. N Engl J Med. (2018) 379:722–30. 10.1056/NEJMoa1805453 30134131 PMC8011001

[7] Vosoughi E Lee JM Miller JR Nosrati M Minor DR Abendroth R. Survival and clinical outcomes of patients with melanoma brain metastasis in the era of checkpoint inhibitors and targeted therapies. BMC Cancer. (2018) 18:490. 10.1186/s12885-018-4374-x 29703161 PMC5924486

[8] Hodi FS O'Day SJ McDermott DF Weber RW Sosman JA Haanen JB. Improved survival with ipilimumab in patients with metastatic melanoma. N Engl J Med. (2010) 363:711–23. 10.1056/NEJMoa1003466 20525992 PMC3549297

[9] Wolchok JD Neyns B Linette G Negrier S Lutzky J Thomas L. Ipilimumab monotherapy in patients with pretreated advanced melanoma: a randomised, double-blind, multicentre, phase 2, dose-ranging study. Lancet Oncol. (2010) 11:155–64. 10.1016/S1470-2045(09)70334-1 20004617

[10] Lehrer EJ Peterson J Brown PD Sheehan JP Quinones-Hinojosa A Zaorsky NG. Treatment of brain metastases with stereotactic radiosurgery and immune checkpoint inhibitors: an international meta-analysis of individual patient data. Radiother Oncol. (2019) 130:104–12. 10.1016/j.radonc.2018.08.025 30241791

[11] Frinton E Tong D Tan J Read G Kumar V Kennedy S. Metastatic melanoma: prognostic factors and survival in patients with brain metastases. J Neurooncol. (2017) 135:507–12. 10.1007/s11060-017-2591-9 28819707 PMC5700221

[12] Sloot S Chen YA Zhao X Weber JL Benedict JJ Mule JJ. Improved survival of patients with melanoma brain metastases in the era of targeted BRAF and immune checkpoint therapies. Cancer. (2018) 124:297–305. 10.1002/cncr.30946 29023643 PMC7771556

[13] Patel KR Chowdhary M Switchenko JM Kudchadkar R Lawson DH Cassidy RJ. BRAF inhibitor and stereotactic radiosurgery is associated with an increased risk of radiation necrosis. Melanoma Res. (2016) 26:387–94. 10.1097/CMR.0000000000000268 27223498 PMC4943024

[14] Diao K Bian SX Routman DM Yu C Ye JC Wagle NA. Stereotactic radiosurgery and ipilimumab for patients with melanoma brain metastases: clinical outcomes and toxicity. J Neurooncol. (2018) 139:421–9. 10.1007/s11060-018-2880-y 29696531 PMC7469981

[15] Chao ST Ahluwalia MS Barnett GH Stevens GH Murphy ES Stockham AL. Challenges with the diagnosis and treatment of cerebral radiation necrosis. Int J Radiat Oncol Biol Phys. (2013) 87:449–57. 10.1016/j.ijrobp.2013.05.015 23790775

[16] Dequesada IM Quisling RG Yachnis A Friedman WA. Can standard magnetic resonance imaging reliably distinguish recurrent tumor from radiation necrosis after radiosurgery for brain metastases? A radiographic-pathological study. Neurosurgery. (2008) 63:898–903. 10.1227/01.NEU.0000333263.31870.31 19005380

[17] Mitsuya K Nakasu Y Horiguchi S Harada H Nishimura T Bando E. Perfusion weighted magnetic resonance imaging to distinguish the recurrence of metastatic brain tumors from radiation necrosis after stereotactic radiosurgery. J Neurooncol. (2010) 99:81–8. 10.1007/s11060-009-0106-z 20058049

[18] Chao ST Suh JH Raja S Lee SY Barnett G. The sensitivity and specificity of FDG PET in distinguishing recurrent brain tumor from radionecrosis in patients treated with stereotactic radiosurgery. Int J Cancer. (2001) 96:191–7. 10.1002/ijc.1016 11410888

[19] Verma N Cowperthwaite MC Burnett MG Markey MK. Differentiating tumor recurrence from treatment necrosis: a review of neuro-oncologic imaging strategies. Neuro Oncol. (2013) 15:515–34. 10.1093/neuonc/nos307 23325863 PMC3635510

[20] Patel KR Shoukat S Oliver DE Chowdhary M Rizzo M Lawson DH. Ipilimumab and stereotactic radiosurgery versus stereotactic radiosurgery alone for newly diagnosed melanoma brain metastases. Am J Clin Oncol. (2017) 40:444–50. 10.1097/COC.0000000000000199 26017484

[21] Kiess AP Wolchok JD Barker CA Postow MA Tabar V Huse JT. Stereotactic radiosurgery for melanoma brain metastases in patients receiving ipilimumab: safety profile and efficacy of combined treatment. Int J Radiat Oncol Biol Phys. (2015) 92:368–75. 10.1016/j.ijrobp.2015.01.004 25754629 PMC4955924

[22] Sperduto PW Jiang W Brown PD Braunstein S Sneed P Wattson DA. estimating survival in melanoma patients with brain metastases: an update of the graded prognostic assessment for melanoma using molecular markers (Melanoma-molGPA). Int J Radiat Oncol Biol Phys. (2017) 99:812–6. 10.1016/j.ijrobp.2017.06.2454 29063850 PMC6925529

[23] Karger CP Hartmann GH Heeg P Jakel O. A method for determining the alignment accuracy of the treatment table axis at an isocentric irradiation facility. Phys Med Biol. (2001) 46:N19–26. 10.1088/0031-9155/46/1/404 11197684

[24] Brasch RC Weinmann HJ Wesbey GE. Contrast-enhanced NMR imaging: animal studies using gadolinium-DTPA complex. AJR Am J Roentgenol. (1984) 142:625–30. 10.2214/ajr.142.3.625 6607656

[25] Chan YL Leung SF King AD Choi PH Metreweli C. Late radiation injury to the temporal lobes: morphologic evaluation at MR imaging. Radiology. (1999) 213:800–7. 10.1148/radiology.213.3.r99dc07800 10580956

[26] Liew DN Kano H Kondziolka D Mathieu D Niranjan A Flickinger JC. Outcome predictors of gamma knife surgery for melanoma brain metastases. Clinical article. J Neurosurg. (2011) 114:769–79. 10.3171/2010.5.JNS1014 20524829

[27] Kocher M Soffietti R Abacioglu U Villa S Fauchon F Baumert BG Adjuvant whole-brain radiotherapy versus observation after radiosurgery or surgical resection of one to three cerebral metastases: results of the EORTC 22952-26001 study. J Clin Oncol. (2011) 29:134–41. 10.1200/JCO.2010.30.1655 21041710 PMC3058272

[28] Mulvenna P Nankivell M Barton R Faivre-Finn C Wilson P McColl E. Dexamethasone and supportive care with or without whole brain radiotherapy in treating patients with non-small cell lung cancer with brain metastases unsuitable for resection or stereotactic radiotherapy (QUARTZ): results from a phase 3, non-inferiority, randomised trial. Lancet. (2016) 388:2004–14. 10.1016/S0140-6736(16)30825-X 27604504 PMC5082599

[29] Lin NU Lee EQ Aoyama H Barani IJ Barboriak DP Baumert BG. Response assessment criteria for brain metastases: proposal from the RANO group. Lancet Oncol. (2015) 16:e270–8. 10.1016/S1470-2045(15)70057-4 26065612

[30] Lwu S Goetz P Monsalves E Aryaee M Ebinu J Laperriere N. Stereotactic radiosurgery for the treatment of melanoma and renal cell carcinoma brain metastases. Oncol Rep. (2013) 29:407–12. 10.3892/or.2012.2139 23151681 PMC3583599

[31] Minniti G Anzellini D Reverberi C Cappellini GCA Marchetti L Bianciardi F Stereotactic radiosurgery combined with nivolumab or Ipilimumab for patients with melanoma brain metastases: evaluation of brain control and toxicity. J Immunother Cancer. (2019) 7:102 10.1186/s40425-019-0588-y 30975225 PMC6458744

[32] Hadi I Roengvoraphoj O Bodensohn R Hofmaier J Niyazi M Belka C. Stereotactic radiosurgery combined with targeted/ immunotherapy in patients with melanoma brain metastasis. Radiat Oncol. (2020) 15:37. 10.1186/s13014-020-1485-8 32059731 PMC7023694

[33] Ahmed KA Stallworth DG Kim Y Johnstone PA Harrison LB Caudell JJ Clinical outcomes of melanoma brain metastases treated with stereotactic radiation and anti-PD-1 therapy. Ann Oncol. (2016) 27:434–41. 10.1093/annonc/mdv622 26712903

[34] Silk AW Bassetti MF West BT Tsien CI Lao CD. Ipilimumab and radiation therapy for melanoma brain metastases. Cancer Med. (2013) 2:899–906. 10.1002/cam4.140 24403263 PMC3892394

[35] Kohutek ZA Yamada Y Chan TA Brennan CW Tabar V Gutin PH. Long-term risk of radionecrosis and imaging changes after stereotactic radiosurgery for brain metastases. J Neurooncol. (2015) 125:149–56. 10.1007/s11060-015-1881-3 26307446 PMC4726630

